# Supplementary material for: Comprehensive analysis of lncRNA-associated competing endogenous RNA network in tongue squamous cell carcinoma
Source: PeerJ. 2019 Feb 6;7:e6397. doi: 10.7717/peerj.6397 (PMC6368841; doi:10.7717/peerj.6397)
Supplement: Table S2 [file peerj-07-6397-s002.docx]

**Supplementary Table S4: Gene ontology analyses of the DEmRNAs according to their biological process, cellular component and molecular function.**

| **Category** | **Term** | **Count** | **FDR** |
| --- | --- | --- | --- |
| **GOTERM_BP_DIRECT** | **GO:0030049~muscle filament sliding** | **25** | **4.11E-13** |
|  | **GO:0030574~collagen catabolic process** | **32** | **4.11E-13** |
|  | **GO:0030198~extracellular matrix organization** | **58** | **8.33E-13** |
|  | **GO:0006936~muscle contraction** | **38** | **3.54E-10** |
|  | **GO:0001501~skeletal system development** | **38** | **1.27E-06** |
|  | **GO:0030199~collagen fibril organization** | **18** | **2.12E-05** |
|  | **GO:0035987~endodermal cell differentiation** | **15** | **2.62E-05** |
|  | **GO:0003341~cilium movement** | **15** | **2.62E-05** |
|  | **GO:0001503~ossification** | **25** | **1.42E-04** |
|  | **GO:0030239~myofibril assembly** | **10** | **3.61E-04** |
|  | **GO:0034220~ion transmembrane transport** | **44** | **4.32E-04** |
|  | **GO:0007155~cell adhesion** | **76** | **4.74E-04** |
|  | **GO:0060048~cardiac muscle contraction** | **17** | **0.001651** |
|  | **GO:0009952~anterior/posterior pattern specification** | **23** | **0.002561** |
|  | **GO:0007267~cell-cell signaling** | **48** | **0.002656** |
|  | **GO:0022617~extracellular matrix disassembly** | **22** | **0.004067** |
|  | **GO:0009954~proximal/distal pattern formation** | **12** | **0.00466** |
|  | **GO:0045214~sarcomere organization** | **13** | **0.006105** |
|  | **GO:0043588~skin development** | **14** | **0.006857** |
| **GOTERM_CC_DIRECT** | **GO:0005576~extracellular region** | **312** | **1.23E-37** |
|  | **GO:0005615~extracellular space** | **264** | **7.68E-32** |
|  | **GO:0005578~proteinaceous extracellular matrix** | **80** | **1.15E-18** |
|  | **GO:0030018~Z disc** | **40** | **5.36E-10** |
|  | **GO:0005581~collagen trimer** | **31** | **4.02E-07** |
|  | **GO:0031430~M band** | **15** | **1.43E-06** |
|  | **GO:0031012~extracellular matrix** | **62** | **1.49E-06** |
|  | **GO:0030016~myofibril** | **16** | **4.16E-06** |
|  | **GO:0042383~sarcolemma** | **26** | **1.23E-04** |
|  | **GO:0030017~sarcomere** | **17** | **1.56E-04** |
|  | **GO:0031674~I band** | **13** | **2.44E-04** |
|  | **GO:0032982~myosin filament** | **11** | **2.73E-04** |
|  | **GO:0016324~apical plasma membrane** | **54** | **0.00121** |
|  | **GO:0005796~Golgi lumen** | **26** | **0.001581** |
|  | **GO:0005859~muscle myosin complex** | **10** | **0.003971** |
|  | **GO:0005788~endoplasmic reticulum lumen** | **39** | **0.005681** |
|  | **GO:0033017~sarcoplasmic reticulum membrane** | **14** | **0.006316** |
|  | **GO:0031093~platelet alpha granule lumen** | **18** | **0.00672** |
|  | **GO:0005930~axoneme** | **22** | **0.009814** |
| **GOTERM_MF_DIRECT** | **GO:0008307~structural constituent of muscle** | **23** | **1.19E-09** |
|  | **GO:0005201~extracellular matrix structural constituent** | **27** | **5.93E-08** |
|  | **GO:0005509~calcium ion binding** | **119** | **8.53E-08** |
|  | **GO:0008201~heparin binding** | **38** | **1.01E-04** |
|  | **GO:0005125~cytokine activity** | **40** | **1.52E-04** |
|  | **GO:0019825~oxygen binding** | **18** | **4.88E-04** |
|  | **GO:0008083~growth factor activity** | **35** | **0.003698** |
