# Supplementary material for: Comprehensive analysis of lncRNA-associated competing endogenous RNA network in tongue squamous cell carcinoma
Source: PeerJ. 2019 Feb 6;7:e6397. doi: 10.7717/peerj.6397 (PMC6368841; doi:10.7717/peerj.6397)

**Supplementary Fig. 1 Heatmaps of the DEmRNAs**


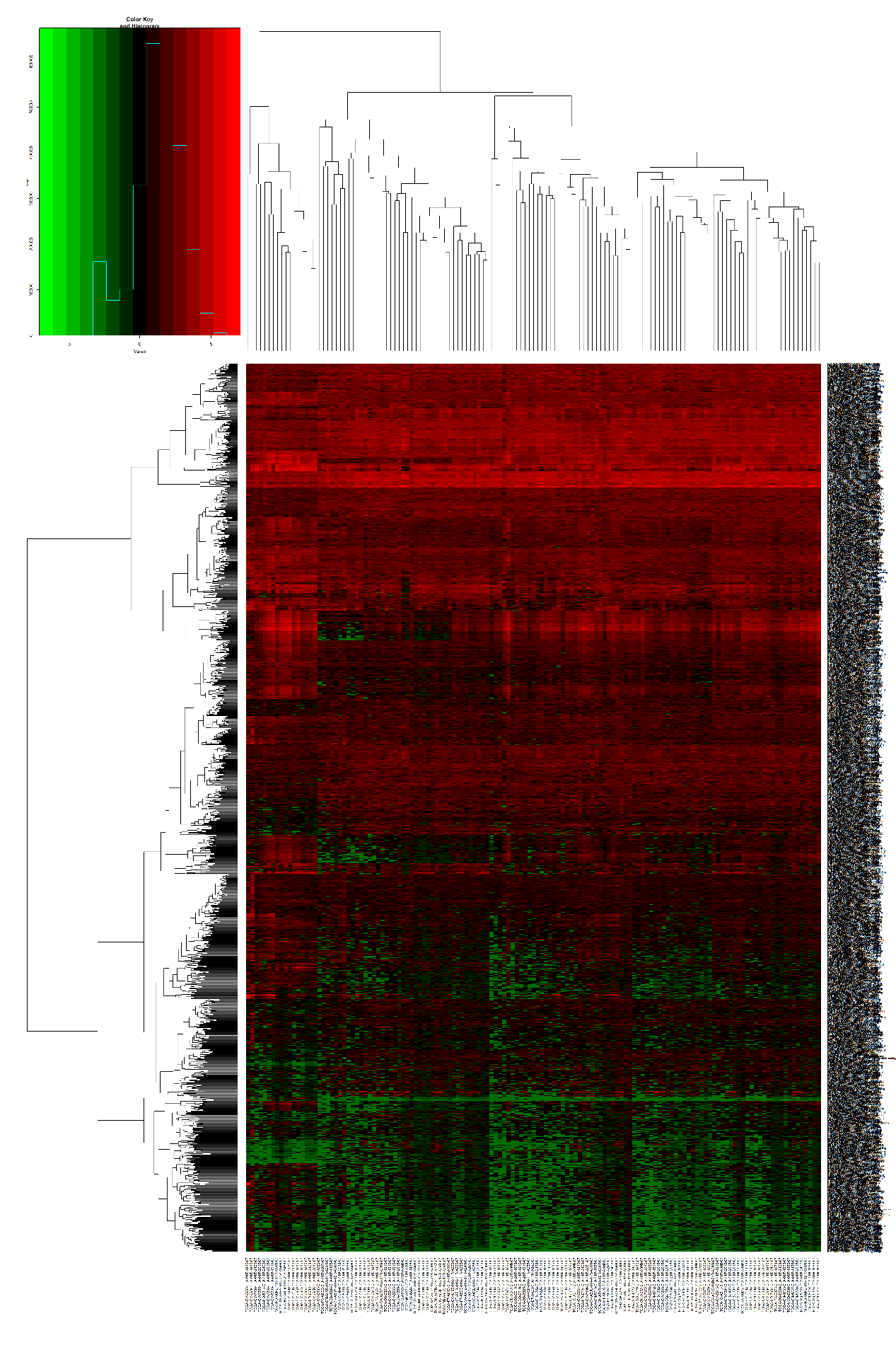


**Supplementary Fig. 2 Heatmaps of the DElnRNAs**


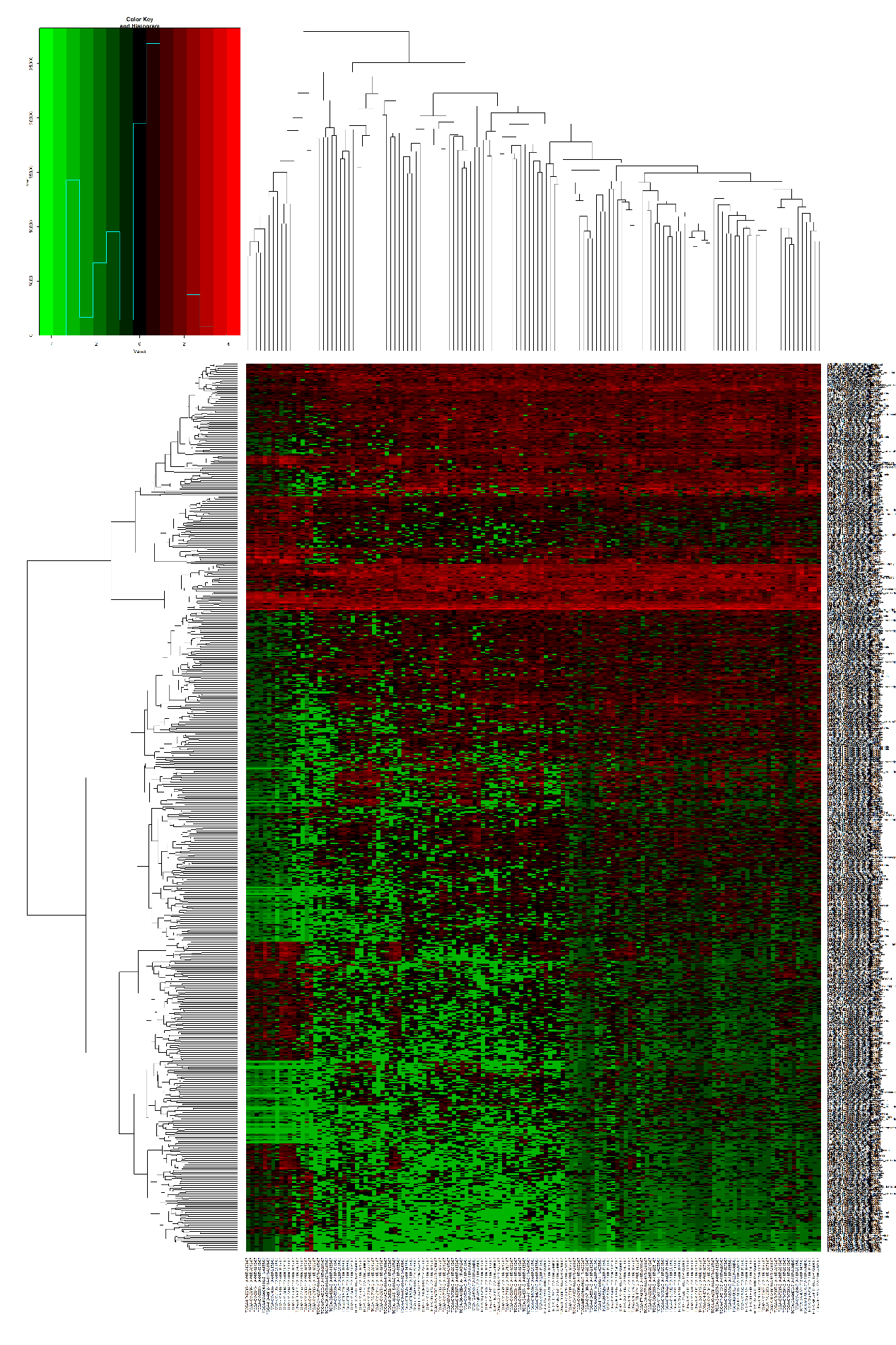


**Supplementary Fig. 3 Heatmaps of the DEmiRNAs**


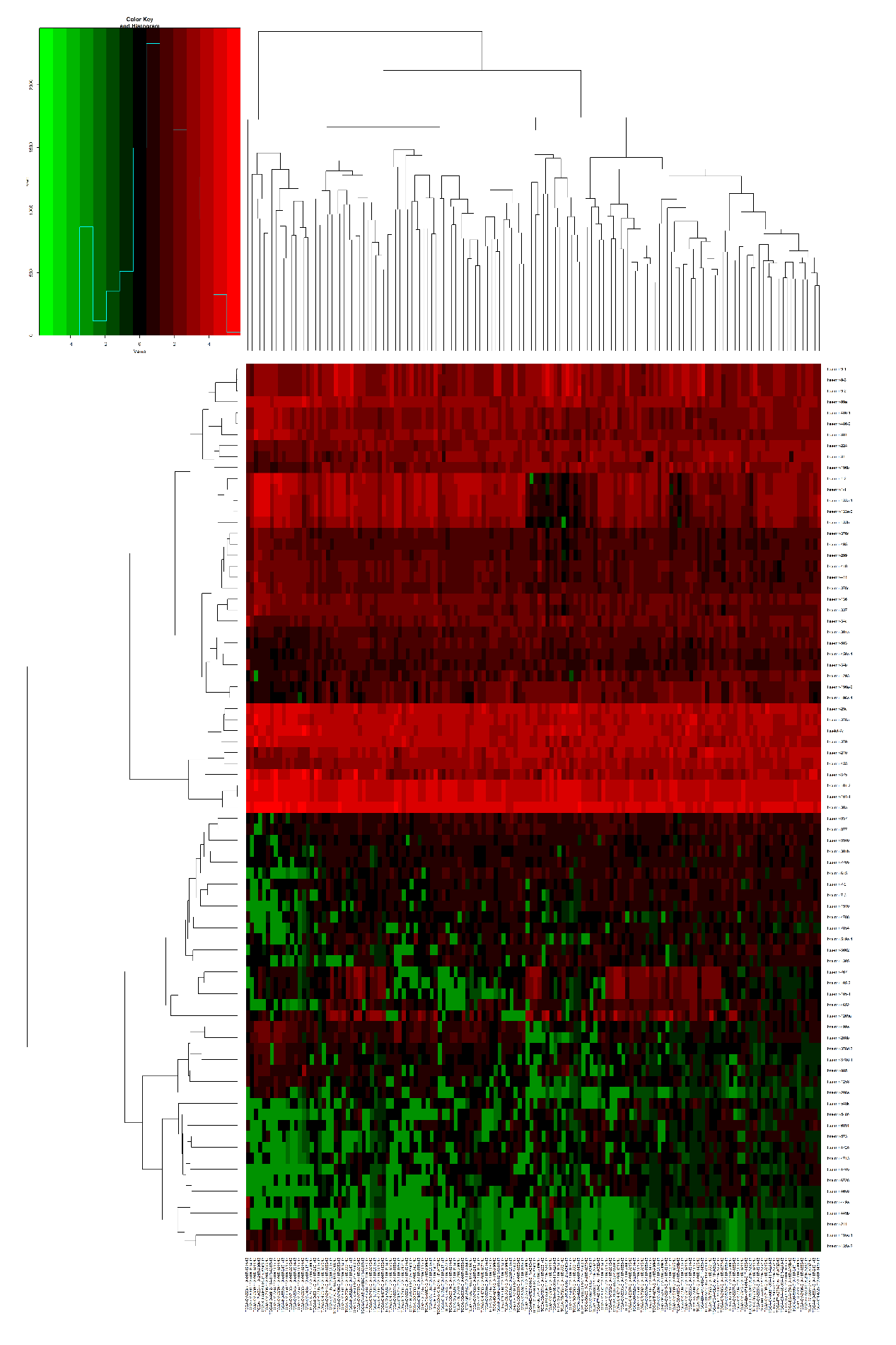

Supplement: Supplemental Information 1 [file peerj-07-6397-s008.docx]
