# Supplementary figures and images for: Comprehensive analysis of lncRNA-associated competing endogenous RNA network in tongue squamous cell carcinoma
Source: PeerJ. 2019 Feb 6;7:e6397. doi: 10.7717/peerj.6397 (PMC6368841; doi:10.7717/peerj.6397)

**Supplementary Fig. 4 Venn diagram of mRNAs involved in ceRNA regulation network.**


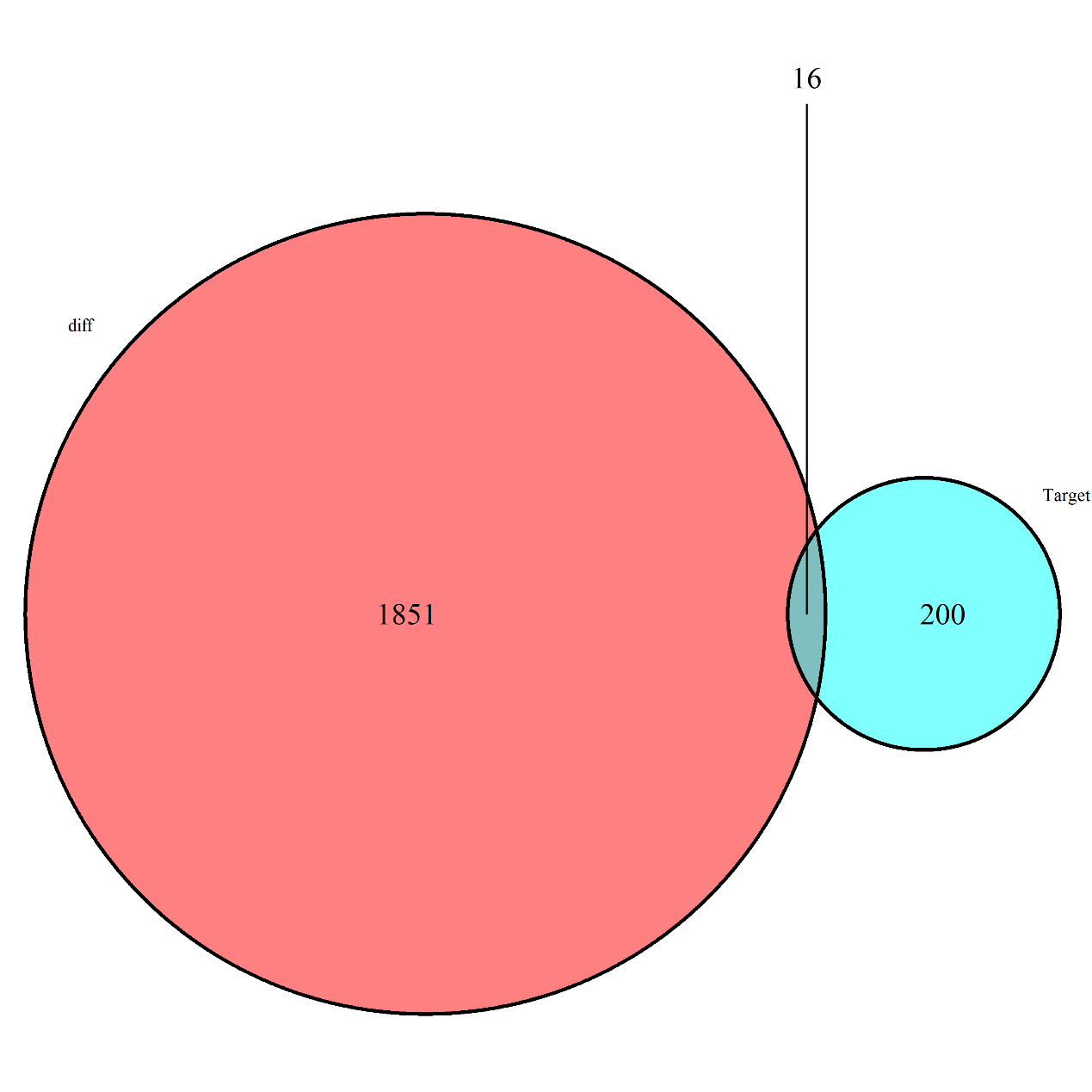

Supplement: Figure S4 — The red area presents only the DEmRNAs number instead of the target number. The blue area presents only the target number, rather than the DEmRNAs number, while the purple area in the middle indicates the number of mRNA which is both the differential expression and the target. [file peerj-07-6397-s009.docx]
